# Action research with caseworkers: Responding to and reflecting on the impacts of COVID‐19 on birth family contact

**DOI:** 10.1111/cfs.12935

**Published:** 2022-05-16

**Authors:** Sarah Ciftci, Susan Collings, Amy Conley Wright

**Affiliations:** ^1^ Research Centre for Children and Families, Sydney School of Education and Social Work The University of Sydney Sydney

**Keywords:** child welfare (in Australia), contact, contact (with birth relatives), COVID‐19, foster care, research in practice

## Abstract

Social distancing due to COVID‐19 forced changes to contact with birth relatives for children in out‐of‐home care. This required a shift to using technologies, which was previously underutilized and viewed as risky. In an action research study, 33 caseworkers in New South Wales, Australia, reflected upon adapting their practices. Three key themes characterized the changes in caseworker practices and how these impacted upon social interactions between children and their birth and carer families: communication, not location; shared not separate spaces and spontaneous not restricted interaction. First, caseworkers described how contact via technologies involved fewer logistical arrangements, shifting the focus on interactions among children and their two families and encouraging these to be flexible and child‐centred. Second, caseworkers discussed how spending time together virtually could build trust, as carers and birth relatives could forge relationships around shared commitment to the child's wellbeing. Third, caseworkers noted that technology‐facilitated communication enabled greater choice and control for children while requiring renegotiating boundaries. The findings reflect a shift in caseworker perceptions of technology‐facilitated contact from a risk to opportunity framework as a result of COVID‐19 conditions, consistent with social shaping of technology theory. Beyond the pandemic, contact with birth relatives can be enhanced through technology.

## 1 INTRODUCTION AND BACKGROUND

The global spread of COVID‐19 prompted a range of responses from governments around the world, including state‐imposed ‘lockdowns’ that limit social and physical contact between individuals. Common social distancing measures include school and workplace closures, bans on public gatherings and stay at home orders (Thomas et al., 2021). Children who live apart from their families as a result of child protection intervention and decision‐making are uniquely impacted by lockdown measures. Lockdowns pose barriers to face‐to‐face visits between children in care and family members they do not live with (Neil et al., 2020). These face‐to‐face visits are an important way in which children's relationships with family members are supported, developed and sustained.

Australian law enshrines the right to ongoing contact with family members for children in out‐of‐home care (foster care, kinship care, guardianship and open adoption), interpreted preferably as direct (face to face) and enacted in court orders or agency‐monitored contact plans (FACS, 2019). This is consistent with a child's right to maintain family relationships and cultural identity as laid out in the Convention on the Rights of the Child (United Nations, 1989) (Art. 9.3, 30) and evidence that children in out‐of‐home care feel a longing for connection to family, kin and community (Biehal, 2014; Cashmore & Paxman, 2006; Sen & Broadhurst, 2011). Yet prior to changes due to the pandemic, a longitudinal study on children's pathways in out‐of‐home care in NSW found that rates of technology‐assisted (or ‘virtual’) contact such as using video calls, emails and social media were low (2–5%) (Cashmore & Taylor, 2017). Given the dominance and preference for direct contact in Australia, using indirect forms of communication was unchartered territory for many children, families and workers alike.

The Australian state of New South Wales (NSW) introduced tough lockdown restrictions in March 2020, which led to the suspension of in‐person interactions between service providers and families and of direct contact between children in out‐of‐home care and birth relatives. The statutory government department responsible for child welfare provided directions for the use of COVID‐safe forms of communication such as phone and video calls and exchange of text messages and emails (NSW Department of Communities and Justice, 2021). This required caseworkers to rapidly adapt their practice to keep children in care and their families connected from a physically safe distance, using commonly available digital technologies.

At the same time as these sudden and necessary changes to children's contact with family were taking place, action research was being used in New South Wales to explore how out‐of‐home care caseworkers could better support children's relationships with their families. Influenced by the onset of the COVID‐19 pandemic, action research teams composed of caseworkers and their managers chose to bring a practice focus to supporting indirect communication between children in out‐of‐home care and their family members. This research provided a unique window to observe practice adaptations by the out‐of‐home care sector in real‐time and to document emerging lessons. After reviewing relevant literature on technology‐assisted contact, this paper will present qualitative, reflective practice evidence from 33 out‐of‐home care caseworkers who met monthly as part of an action research study while adapting their practice during COVID‐19 lockdowns and restrictions.

### 1.1 International evidence on technology‐assisted contact

The use of mobile devices and internet‐based digital technologies has become an integral part of contemporary life, and, as such, the potential for digital technologies to impact the nature of contact between children in care and their families is receiving growing attention. A number of international studies, primarily in the UK, have examined the views and experiences of children, carers, family members and social work practitioners in relation to using digital technologies for contact. Various terms are used to describe this contact, including indirect contact, technology‐assisted contact, digital or virtual contact and family time from a distance.

The small but growing body of literature on digital contact between children in out‐of‐home care and their family members suggests it is not possible to conclude that digital contact is positive or negative (Iyer et al., 2020; Neil et al., 2020). A rapid evidence review undertaken in the UK in response to COVID‐19 related social distancing measures explored the implications of digital contact on children separated from their birth relatives, synthesizing findings from 16 international studies in both public and private law contexts (Iyer et al., 2020). A contemporaneous UK study used an online survey (*n* = 197) and telephone interviews (*n* = 24) with carers, professionals and birth relatives to investigate what was working well and not so well in terms of contact between children in or adopted from care and their families during lockdowns (Neil et al., 2020). Cumulative findings suggest that digital contact experiences are unique and involve both challenges and benefits (Neil et al., 2020). For example, digital contact can be more immediate and less formal and may help facilitate relationships, but having meaningful and positive interactions can involve active support to manage expectations and transitions, set boundaries and be age appropriate (Iyer et al., 2020; Neil et al., 2020). Taken together, this evidence echoes existing research on the benefits of direct contact, which suggests there is no simple, one‐size‐fits‐all approach (Boyle, 2017). These studies highlight the need to apply best practice principles for children's contact with families, regardless of the mode of communication used. These include taking a child‐centred and child‐friendly approach, considering the needs and wishes of both families and making contact arrangements work on an individual, case‐by‐case basis.

Research reveals that perspectives on the benefits and risks of digital contact diverge. On the one hand, children and parents/relatives are more likely to view technology‐based contact primarily as an opportunity for informal family connection. Simpson (2020) studied 12 triads of young people, their foster carers and social work practitioners to explore how young people use mobile communication devices and internet for contact with people they value, including family. Young people described digital contact as a means of ‘staying in touch’ informally and spontaneously with family members, rather than ‘contact’, which they characterized as formulaic and rigid arrangements and interaction patterns (Simpson, 2020). While limited research exploring the views of birth relatives in relation to digital contact exists, one study of 62 parents of children in foster care highlights the value parents place on opportunities to connect with their children by mobile phone when direct contact is limited or supervised, including being able to text or call at regular times, such as at bedtime (Schofield et al., 2009). By contrast, carers and practitioners are more likely to perceive it as a risk to children's safety and wellbeing. In Simpson's (2020) study, for example, carers and social workers did not see the value of technology‐assisted contact for supporting ongoing family relationships, framing it as a ‘risk or nuisance’ rather than an opportunity for relationship‐building and a normalized communication mode for young people.

This sentiment is echoed in the wider literature on attitudes towards young people's use of technology in which carers, adoptive parents and social workers emphasize safeguarding from online harm, risk management and the perils of overusing devices (MacDonald et al., 2017). Moreover, digital contact is viewed as posing significant risks to the emotional wellbeing of children and young people when it occurs ‘out‐of‐the‐blue’ or outside of an established relationship; it has been formally prohibited or is unsupported by carers and adoptive parents (Greenhow et al., 2016; MacDonald & McSherry, 2013; Neil et al., 2013). Yet it is important to note that a study by Sen (2010) found that even when practitioners did not have first‐hand experience of children using technology for contact, they constructed it primarily as a risk rather than an opportunity.

Despite the dominance of risk discourse, research nonetheless points to the value of technology‐assisted contact for extending and developing existing relationships between children and their families. Virtual contact is thought to add ‘normality and reality to the child's dual connection to two families’, allowing the connection ‘to feel more natural and family‐like’ (Greenhow et al., 2016, p. 382) through its informal nature and regularity (Neil et al., 2013, p. 245). Willoughby (2019) stresses that practitioners need to build their knowledge and skills with technology‐facilitated communication and social media in order to reduce risks and promote opportunities for young people and so they can offer a supportive scaffold for all parties involved. The need for further research into the views and concerns of social work practitioners around technology‐assisted contact has also been identified, in light of the central role they play in managing contact arrangements (Sen & Broadhurst, 2011, pp. 305–306).

### 1.2 Social work and the social shaping of technology

Such calls are particularly pertinent given that the social work profession had been notoriously slow to use technologies and develop technology‐facilitated practices even prior to the COVID‐19 pandemic (Carrilio, 2005; Taylor‐Beswick, 2019). This is despite a wealth of evidence pointing to the relevance of technologies for relational and participatory social work practice, including opportunities to enhance the active participation of children and families in child welfare processes (Tregeagle, 2010) and the potential for technology‐mediated communication to assist social workers and service users to develop *social presence* – or an awareness of the other person (LaMendola, 2010; Simpson, 2017). The theory of technological essentialism posits that technologies direct social change and their uptake is inevitable and positive (Hutchby, 2003), claims which are challenged by the low uptake within the social work profession. In contrast, a social constructionist view of technology and accompanying ideas of social shaping of technology posits that technology is shaped by complex social processes and social factors (Hutchby, 2001). Like Tregeagle (2016), we argue that the use of technology is ‘both the result of, but also shapes, social processes’ (p. 228) and that the COVID‐19 pandemic is a factor that shapes uptake and use of technology.

The research presented here extends on this body of literature through an analysis of caseworker reflections about their own practices to support children and families to connect from a physical distance during COVID‐19 restrictions. The study sought to answer the following two research questions: (1) How did caseworkers support children, carers and birth family to have indirect contact? And (2) how did caseworkers view the use of technologies to keep children connected to family during COVID‐19?

## 2 METHOD

These findings are part of a larger study, *Fostering Lifelong Connection for Children in Permanent Care*, that uses action research to develop, trial and evaluate small practice changes to improve relationship‐based casework support for contact (referred to in the study as ‘family time’) over a 2‐year period. Participatory action research is a qualitative methodology designed to promote, document and evaluate a change process (Chevalier & Buckles, 2019; MacDonald, 2012). It involves critical observation of practices and concepts and flexible responses to lessons as they emerge (Reason & Bradbury, 2001). This method acknowledges that people who have personal experience of an issue have the knowledge that is critical to designing workable solutions to it (Chouinard & Milley, 2016; McCormack & Dewing, 2012). Action research participants engage in a cycle of planning, acting, reflecting and adapting (Cardno, 2003; McNiff & Whitehead, 2012). The study adapted an action research methodology, the Breakthrough Series Collaborative, that was first developed in health settings and then utilized in the context of child welfare practice in the United States to bridge the research‐to‐practice gap (Miller & Ward, 2008). The first of three action research cycles of the study commenced in March 2020 and coincided with the onset of the COVID‐19 pandemic. The action researchers brought intentional focus on practices to support children and their two families to engage in family time from a distance. Ethical approval for the study was obtained from the University of Sydney Human Research Ethics Committee.

### 2.1 Sample and participants

A purposive sampling method was used to recruit 33 caseworkers and their managers from eight out‐of‐home care organizations, each with children on their caseloads who were permanently placed in out‐of‐home care, on court orders for long‐term foster care or kinship care or transitioning to open adoption or guardianship and having regular contact with birth relatives (Table 1). The overarching aim of the action research was to improve practices used for family time through building practice‐informed evidence, and the professionals who are ‘information rich’ (Patton, 2002) in this practice area are caseworkers. This was an efficient and practical approach to selection of individuals with relevant knowledge (Cresswell & Clark, 2011), willingness to participate and ability to communicate experiences in a reflective manner (Palinkas et al., 2015).

**TABLE 1:** Participant details

Participant details	No. of participants (%)
Gender	
Female	33 (100)
Male	0 (0)
Aboriginal and Torres Strait islander	
Yes	3 (9)
No	30 (91)
Role	
Caseworker	(67)
Casework manager	11 (33)
Organization type	
Government	12 (36)
Non‐government	21 (64)
Location	
Urban	18 (55)
Regional	15 (45)
Total	33

### 2.2 Data collection

Data collection took place between March and October 2020. The 33 action researchers formed four site‐based local action research teams and participated in reflective meetings in their local teams each month. Reflective practice is a core concept in social work owing to Schon's (1983) formulation of how professionals engage in ‘reflection *in* action’ by thinking consciously about what they are doing while they are doing it and later using ‘reflection *on* action’ to integrate their practice with theory and knowledge (Fisher & Somerton, 2000; Ferguson, 2018). These meetings were attended by at least one member of the academic research team and were audio recorded. If an action researcher was unable to attend, they submitted a brief, written reflection. Action researchers were invited to reflect on what worked well and not so well during the previous month, challenges and how they overcame them, new learnings and impacts on children's relationships. Due to COVID‐19 restrictions, meetings were mainly convened on videoconferencing platform Zoom and audio recorded to a local drive of a laptop before being transferred to a secure data store and permanently deleted from the local drive. This protected the privacy of participants. Audio recordings were transcribed for use in analysis.

### 2.3 Data analysis

An inductive approach was used to analyse the reflective data using a six‐phase process outlined by Braun and Clarke (2012). Data were uploaded to Dedoose™, a secure cloud‐based program designed for mixed method, team‐based research. Author 1 completed open coding of all data and proposed preliminary categories and themes. Authors 2 and 3 completed inter‐rater reliability tests of a sub‐sample of codes from each theme in Dedoose™. The authors iteratively resolved discrepancies in interpretation through discussion. Exemplar themes were re‐examined to remove duplicates or rename constituent codes or categories. The codebook was applied, tested and revised in an iterative process until a 0.80 kappa score of inter‐rater agreement was achieved among the three authors.

## 3 FINDINGS

Caseworkers across the four action research locations told us responding to the rapid and unexpected changes introduced by agencies due to the COVID‐19 pandemic gave them permission to challenge conventional approaches to family time practice. Prior to COVID‐19, casework time was consumed by complex negotiations with carers and birth relatives about logistics such as duration, timing and location of visits. The three themes of communication, not location, shared not separate spaces and spontaneous not restricted interaction explore practice changes that occurred when caseworkers reimagined social interactions between children and family members as free from the limits imposed by face‐to‐face visits.

### 3.1 Communication not location

This theme explores the way that family time from a COVID‐safe distance prompted caseworkers to shift their focus away from where visits took place and to take creative licence and calculated risks with trialling new modes of communication that can help connect children with relatives. What they observed was that relationships could continue and even flourish in the absence of physical contact when an intentional focus was to create the conditions for meaningful exchange. However, this was not without its challenges.

#### 3.1.1 Focus on quality interactions

Caseworkers reflected that a focus on family time logistics could be a distraction from the fundamental purpose – to create a space for a meaningful connection between children and their relatives to flourish – and undermine efforts to make this as high‐quality experience as possible. When organizing in‐person visits, caseworkers, children's carers and birth relatives often planned for stimulating, activity‐based experiences, for example, going to a play space.

The effort needed to accommodate everyone's preferences and ensure children's physical safety which inevitably often led to less imaginative practice and favoured convenience over quality. It was commonplace for visits to be held at the same location every time, such as playgrounds, playcentres, fast food outlets and shopping centres. While these arrangements may have adhered to the contact plan, it meant visits could become monotonous and bear minimal resemblance to the stated aims of supporting children to sustain healthy relationships with family.

Bearing witness to the difficulties many children and families had with engaging over technology drove home the shortcomings of a standard, activity‐driven style of face‐to‐face visits. When this overemphasis on planning activities was removed, caseworkers reflected that their focus should be on supporting connections, rather than the logistics of spending time together. This is summed up by the following observation:

I think for me it highlighted that some of the relationships that I thought were really strong aren't as strong as they should be and that face‐to‐face visits were definitely overrun by fun activities and doing rather than connecting … I've learnt that there is definitely more work that can be done in the future regarding building a truer connection between children and their parents.

Family time organized via technologies concentrated the attention on communication and interaction among children and their two families. One caseworker explained that it was important not to undervalue the quality of interactions that occurred using technologies just because they may be shorter:

I've been at their face‐to‐face contacts that go for three hours and [15 minutes is] probably equivalent to how much they would talk because they play in the playgrounds. There's even probably more substance to it now to be honest. So, it's quality over quantity.

#### 3.1.2 Familiarity and comfort with digital communication

Although technology is now an integral part of everyday life for most families, prior to COVID‐19, it had not been consistently or widely incorporated into out‐of‐home care practice as a mechanism to support children to stay in touch with family members. Caseworkers recognized that the majority of children and young people they worked with have grown up in the era of digital communication and it is '*their medium'*. For many, using mobile devices and consoles for solo or group play games and social networking sites for maintaining relationships makes the digital world *a 'comfortable space to be in'*. A common observation made by caseworkers was that (school‐aged) children generally found it easier than adults to accommodate the transition to technology‐mediated communication for family time interactions because ‘it seems like a natural thing for them to engage with family and caseworkers this way’. For adult family members and, indeed, many caseworkers, it took more work to adapt.

Spending time with family members at a physical distance had the added advantage for some children and young people of being even more comfortable and emotionally safe, particularly those who usually experienced distress before or after visits. As one participant stated ‘I think it's a lot to do with the environment. They're in their home, with their carers, with their stuff and there is that bit of safety with distance’. When children and young people felt comfortable and more relaxed, positive interactions were more likely for all involved. Indeed, it could provide the conditions needed to help children overcome emotional hurdles to seeing family members in person. A caseworker explained,

One of our kids … had an unplanned, quick 10‐minute [video call] with his mum and we are going to be looking at doing that for him in the future because that was a way of him having contact that he felt more comfortable with. He still does not want face‐to‐face [contact], but he's happy to have a phone call or a [video call].

#### 3.1.3 Improving communication takes time

Children and adults with limited or no experience using digital technologies to communicate found it more difficult than others to adjust to interactions using technologies, but caseworkers reported that even some families who were technologically literate struggled to maintain conversations. As is the case with supervised visits that do not go well, awkwardness and silence can allow tension and fear to creep into the interaction. This can be easily misinterpreted as disinterest or rejection over a screen or by phone and, if a scheduled family time interaction is cut short by children and young people, family members are often left feeling disheartened.

Caseworkers needed to overcome an initial perception by some adults that if you were not sitting next to your child at the cinema or kicking a ball to them in a park you were not really joining in a shared activity. Caseworkers reflected on what made interactions best for children at physical visits that ‘when they are face‐to‐face they have an activity that preoccupies them’. The quest for caseworkers, then, was to come up with creative ways to replicate the positive aspects of face‐to‐face visits in the digital environment. The research team worked with caseworkers to design tip‐sheets for families, for conversation starters and ideas for activities that could be easily done using technologies, such as playing online card games or reading a picture book on a video screen. A caseworker shared how she had helped two brothers, aged 9 and 13, to make conversations with their parents over video chat less stilted and awkward.

We developed a plan together that they would organise to play a card game with their parents. I e‐mailed mum and dad and asked that they get a pack of cards ready for their next [video call] as the boys had developed their own individual card games they would like to teach and play with them. The outcome of this was positive and [they] were able to interact and have fun.

Over time, caseworkers reported that children and their family members were able to interact for longer and need less prompting from caseworkers and carers. The provision of casework support and a chance to try out different forms of technology led to ‘more parents … taking up the option of [video] calls’. Providing children and family members with practical tips to help gradually extend their conversations made a difference to the satisfaction and comfort they felt about interacting by phone or video chat:

At first it was just a quick 5 minute catch up and that's all they could manage because the kids were struggling and then they were given more support … they were given some prompting questions so they could keep the conversation going a bit longer the next time. Now we are up to 13 minutes!

#### 3.1.4 Adapting communication to the child

Caseworkers immediately recognized that it was critical to match the most suitable technology‐based communication to the child's age and developmental stage. Younger age or less developed verbal communication skills make it harder to ‘keep [children's] attention and engage with parents’. Caseworkers actively encouraged carers and family members to trial video calls even with very young children and children with communication disorders. As the following example shows, video calls allow family members to observe their child at play and read their body language.

She's only two and she does not talk and I was trying to give [carer] ideas like maybe you could put the camera sitting up against something so dad can just watch the child play … so he's getting that interaction.

Caseworkers explained to family members the advantage of video calls over phone calls for children with disability. They assisted parents to interpret children's behavioural cues, thereby improving their understanding of their child and ability to engage with them.

I said to mum ‘look, you really have to be able to gauge his reactions and you cannot do it on phone calls’. With the [video call], if she was losing him, she could redirect and now [restrictions have eased], they are actually spending more time face‐to‐face because she's getting his cues.

### 3.2 Shared not separate spaces

This theme explores how technology‐mediated communication allowed children and their two families to bridge the physical space that separated them and to inhabit a virtual space together. Spending time in this space could help build or repair emotional and psychological safety and comfort.

#### 3.2.1 Building trust between adults

The pandemic‐induced changes created new opportunities and ‘almost forced people – the parents and carers – to have to build that relationship in order to have any contact’. Caseworkers explained that supervised face‐to‐face visits had often left carers feeling in the dark about what had taken place, so an advantage of using technologies to facilitate visits was that it allowed carers to see and hear family members engage with their children. As one caseworker put it, ‘all that time she's fretting about what mum's been saying whereas with [video call] it's all open, she's actually been able to see the interaction’.

Similarly, birth relatives often feel they know nothing about their child's life outside their in‐person visits, so staying in touch via technologies between visits helped them feel more involved in their child's everyday life. Video chats gave them relatively more immediacy and access to their child's home life and a chance to see them interact with the carer, which could reassure them that their child was safe and well cared for. One caseworker describes how important this was for a mother whose children had only recently gone into care.

She was finding it difficult, but I think after that video call, she felt really happy with where the kids were living. She could see the dynamic with the carers and the kids was going well. She had lots of positive things to say about the carer and how they were living.

The prospect of meeting each other for the first time can be daunting for carers and birth relatives. Having more exposure to one another in a digital environment helped build familiarity between birth relatives and carers and challenge preconceived or negative beliefs about each other. As one caseworker put it, ‘by trusting everybody and everyone feeling like they were trusted, it changed everything’. For birth relatives, another benefit of video chat was seeing that carers wanted to help them to strengthen their connection with their child: ‘the carers have been able to see how much he enjoys that relationship with mum and they feel really comfortable about moving forward’.

#### 3.2.2 Foundation for the future

Being able to gain some sense of familiarity from a physical distance also laid the foundations for a more relaxed in‐person meeting after restrictions were lifted because it ‘alleviates some of that anxiety’. For example, a carer had met the child's birth father for the first time during virtual visits because she needed to actively facilitate the child's interactions with him. As the caseworker explained, ‘now she's actually coming along to the face‐to‐face visits. I hope that by next year she'll be supervising them’.

Caseworkers also observed a noticeable improvement in the relationships of some carers and birth relatives who did not get along before the lockdown due to their increased contact with one another over video technology. A caseworker explained how technology‐mediated communication had mended a damaged relationship between a maternal kinship carer and paternal family member:

They did not really get along and it was quite hostile [but] since we have moved to [video calls], phone calls and things like that, they have had more exposure [to each other] and … they are now organising visits outside of the schedule, and they are quite happy to even do visits in the home as well to make the little one more comfortable.

### 3.3 Spontaneous not restricted interactions

This theme explores the differences that using technologies made to the way that children and family members communicated and the ways that caseworkers supported them to embrace the benefits and navigate the challenges.

#### 3.3.1 Choice and control

Increased spontaneity was an advantage of the shift to family time from a distance during COVID‐19. Some children and young people were able to gain flexibility and control by asking their carers to initiate phone or video calls rather than having to wait for a scheduled visit. It also made it more acceptable for carers and children to exchange contact details and social media profiles with family members and, in the case of young people, to directly initiate communication with relatives. Caseworkers reflected that some children and young people appeared to be more settled as a result because ‘They have the ability to contact their parents whenever they want. No one is saying “no, you have got to wait until next week” or “you have to have prearranged phone calls”’.

Another caseworker highlighted how the option of using technologies to stay in touch with family was giving children more of a voice in when and how they speak to family, shifting control over family time from agencies and adults to children and young people:

I think with the [video calls] too, instead of having the schedule, it's actually when the kids want to talk and have something to say. So, the conversations are a bit more meaningful rather than ‘today at 6 o clock, you have got to ring mum’ …. The spontaneity helps. They can do it right there, right now, instead of waiting a month and they have forgotten about it.

With the spontaneity offered by technologies and less emphasis placed on sticking to a pre‐determined schedule, caseworkers reported higher frequency and regularity of contact between children and families: ‘in the past, the children may have been seeing family members four to six times per year. Currently, they are having regular, sometimes weekly, phone calls’.

#### 3.3.2 Navigating boundaries

During the transition to family time from a distance, caseworkers were aware of the need to navigate changed boundaries between carer and birth families. Some carers expressed reservations about disclosing personal contact details to family members to allow the child to maintain contact with their family. Caseworkers were trying to quickly familiarize themselves with a new world of online videoconferencing platforms and determine which ones afforded ease of use and flexible user settings. As time went on, caseworkers gained confidence in their ability to safeguard personal contact information and balance the needs of children and families. In some cases, this was by providing parents and carers with a different sim card to use specifically for family time. As one caseworker put it, ‘It's just about trying to figure out what's safe and being flexible’.

Some carers were concerned about the increased volume of video and phone calls children were receiving from birth relatives. Having a schedule for phone and video calls also helped re‐establish boundaries when children gave their phone numbers to birth relatives without carer knowledge. A caseworker explained to the carer, ‘it's okay if you do not respond immediately … When you are free, then you can call mum back’. Young people may want to express their independence by taking charge of how and when they communicate with family. It was up to caseworkers to reassure anxious carers that this was a normal part of adolescent development and to actively help young people and their families to reach an arrangement that worked for everyone.

In other cases, the promise of having an immediate connection by phone or video calls was not realized. Caseworkers heard from carers about children feeling disappointed and rejected after repeated attempts to reach family members were unsuccessful. In response, caseworkers helped the adults establish rules for communication exchange and offered carers guidance on how to deal with unexpected or excessive incoming calls from family members.

Some caseworkers said that it was initially a challenge to help children's birth families to navigate conversations on video calls. Caseworkers were on call to provide constructive feedback during and after video chats to help adults learn how to initiate suitable conversation topics with children. As one person explained,

We started them off with having the phone on loudspeaker but it's now to the point where the parents are quite aware of certain things that they should hold back from saying like ‘you are going to come home’.

When conversations did stray into inappropriate territory, caseworkers were careful to reiterate to carers the gains for children of using technologies to stay connected to family and to normalize the inevitability of boundaries being crossed from time to time, irrespective of whether family time was in‐person or not.

### 3.4 Benefits outweigh drawbacks

Overall, caseworkers viewed technology‐mediated communication as having more advantages than drawbacks. The shift to distant family time during COVID‐19 prompted caseworkers to critically reflect on the rigidity of past contact practices and the impact of this on relationships between children, carers and families. One caseworker described her hopes for a more flexible attitude towards family time:

I kind of want to remove the rule of ‘okay you only speak to each other once a month at this time’. I would like to not see it like that anymore and I'm hoping [carers] come along for the ride … and give the children the opportunity if they want to ring the parents, they can ring them at any time.

Caseworkers began to re‐evaluate their practice and look forward to how these changes could be sustained after restrictions were lifted. They emphasized the value of technologies to keep children and their family members connected in‐between face‐to‐face visits and discussed how they would aim to prioritize this in the future as a supplement to face‐to‐face visits. Another caseworker said,

I'm going to aim to incorporate some of that informal connection into family time … really encouraging the carers to liaise with mum and dad by email or whatever … to I guess make that connection a little bit stronger between face‐to‐face visits.

Caseworkers demonstrated a commitment to shifting their practice, stating for example that ‘we have shown that the [video calls] and those types of things work … to be able to add those in‐between visits is a bonus’.

Caseworkers suggested that their reflection in action while adapting their practice during lockdowns had fuelled organizational learning. They noted how at an organizational level, prior to the COVID‐19 pandemic, ‘we have baulked at that, using the technology, around security and safety’ and how the experience had ‘highlighted our underutilised use of technology’. The need for caseworkers to modify their family time practice provided a transformative learning opportunity for out‐of‐home care organizations. As one caseworker described,

From [our organisation's] perspective … We've probably discounted doing that video call type format before and not seen the value and not seeing that as being good, hearty contact whereas now our views have definitely changed on that … I think COVID has obviously had lots of negatives but from this perspective, it's been a real positive. We've been able to keep kids connected and, in fact, with some kids, get them more connected.

## 4 DISCUSSION

The social shaping of technology perspective draws attention to the agency people hold in determining how technology is used, focusing on cultural factors such as perceptions and meanings (Baym, 2015). The caseworkers in our study were able to move beyond well‐documented, pre‐pandemic risk‐averse perceptions of technology‐mediated contact towards a recognition of the connectedness it can offer between children in care and their families. The COVID‐19 pandemic was described by caseworkers as creating the conditions necessary to enable this shift in perception – a social factor that shaped the way technology was utilized and implemented. It seems rapid and necessary pandemic‐related practice change, coupled with reflection in action allowed for ‘overturning previous complacency and taken‐for‐grantedness of (what used to be) everyday life’ (McDermott, 2020, p. 1), including what other research has reported as the dominance of a risk and safeguarding discourse in the views and attitudes of social work practitioners regarding the merits of technology‐assisted contact (Macdonald et al., 2017; Sen, 2010; Simpson, 2020).

The digitalisation of contact during COVID‐19 lockdowns was not without its challenges. Caseworkers reflected about the time it took for all parties to adapt, difficulties participating in technology‐assisted contact depending on age and developmental stage and the support needs of children and families with regards to meaningfully interacting with one another in a virtual setting while maintaining established boundaries. Yet caseworkers largely reported that the benefits outweighed risks and difficulties. We posit that this owes to a determination caseworkers possessed to ensure children remained connected to their family members despite the physical distance put between them by the pandemic, rather than the mere existence and availability of technologies to support contact. The findings reflect how caseworkers and the children and families they work with responded to technologies and modified their perceptions and behaviours within a COVID‐19 social context, reformulating the way contact is understood, in line with the posits of social shaping of technology (Baym, 2015).

Through new experiences of supporting children to connect with their families from a COVID‐safe distance, caseworkers learned that communication through mobile technologies and social media could foster connectedness and a sense of belonging for children and young people in care, consistent with the aims of family time. Licoppe's (2004) concept of ‘connected presence’ posits that relationships are grounded in continuous and recurring interactions that are both co‐present (face‐to‐face) and mediated (using technologies). Drawing on the work of Licoppe, Christensen (2009) describes how mediated communication practices are integrated into modern family and everyday life, creating an experience of ‘closeness’ and ‘presence’ between family members and assisting families to overcome temporal and spatial dispersion. Simpson (2020) suggests that the immediacy and reach, variation in duration and choice and control afforded by technology‐assisted contact for young people in care contributes to ‘connected presence’. While the focus of family time practice in Australia has been on co‐present interactions, the findings of our study show an optimistic embracing of mediated communication by caseworkers owing to the social conditions produced by the COVID‐19 pandemic. During this time, caseworkers expanded their perspectives of contact, acknowledging the capacity for mediated communication to reaffirm and reactivate children's relationships with their families, allowing them to remain close when they were physically apart. They likewise noted how the immediacy and frequency with which children could communicate with their family members challenged conventional family time practice which is characterized by scheduled timing, duration and location of face‐to‐face visits. As such, we argue that an interplay of influence exists between pandemic conditions and caseworker perceptions of digital technologies, including how caseworkers create meaning about their benefits and use (Kretchmer, 2018).

Another example of this interplay is evident in the research results that indicate that technology‐assisted, indirect contact can assist with developing ‘communicative openness’ between carers and birth family members. In adoption contexts, openness relates to ‘the degree to which information passes between birth and adoptive families and to the level of contact and ongoing relationship between these connected families’ (Jones & Hackett, 2007, p. 158). Neil's (2009) work on communicative openness in adoptive families describes communication with the birth family, comfort with and promotion of dual connection and empathy for the birth family as key components. In NSW and indeed Australia, the emerging model of child welfare policy and practice emphasizes the need for *permanency with lifelong connections to birth families* (Wright & Collings, 2019). Children's ongoing relationships with family are prioritized regardless of the placement type they are in. Previous studies have shown that technology‐assisted contact works best when a ‘general climate of openness’ within adoptive families already exists (Neil et al., 2013, p. 244).

Our study extends on these findings, suggesting that technology‐assisted contact can be a means for developing and facilitating greater openness between carers of children in out‐of‐home care and their birth relatives in circumstances where openness is yet to exist. The results indicate that video calls gave carers and parents who had not met a chance to observe each other from a comfortable physical distance and could make them more receptive to in‐person contact in the future. Again, these findings reflect how the use of technology is socially shaped and reciprocally shapes individuals, groups and society (Kretchmer, 2018). As the use of technologies for family time became normalized during pandemic restrictions, so too did the practical necessity for children, carers and birth relatives to exchange contact details and social media profiles. Caseworkers witnessed the resultant strengthening of communication and levels of information shared between carer and birth families. This was a marked practice shift from pre‐COVID‐19 times where agency supervision and mediation of communication were the norm unless carers facilitated contact, usually in preparation for guardianship or open adoption orders. Removing another layer of agency oversight and mediation between children's two families created a chance for collaboration and relationship‐building between carers and birth relatives in the out‐of‐home care context.

Best practice approaches for supporting children in care and their families to communicate via technologies requires further attention and development, including in relation to protecting user privacy. Given pre‐pandemic social work was less reliant on digital technologies and social work education was less willing to prepare its workforce in digital capability, investment should be made in developing these skills across the profession to enable critical digital decision making (Taylor‐Beswick, 2021).

## 5 LIMITATIONS

The study relied solely on caseworker accounts of how children and families experienced the shift to non‐physical modes of communication and the suitability and success of casework responses. Consistent with an action research approach, the academic researchers made no attempt to verify the accuracy of the accounts or to challenge how individuals interpreted their observations. However, data collection at regular intervals with the same caseworkers over a 6‐month period provided an opportunity to follow up on previously noted observations and clarify inconsistencies, thereby enhancing reliability. The co‐researcher sample was recruited using a convenience approach which may have introduced recruitment bias. While it is recognized that the co‐researchers involved in this study are not representative of all caseworkers in NSW and group and individual differences may have influenced their accounts, the impact of these differences on generalizability of results is reduced by a study design that included caseworkers from government and non‐governmental organizations and from urban and regional parts of the state. Finally, input was not sought from children or family members about the practices trialled by caseworkers, and data triangulation would have strengthened the reported results.

## 6 CONCLUSION

The need to rapidly respond to unexpected changes due to the COVID‐19 pandemic gave caseworkers permission to challenge conventional and risk‐ oriented approaches to family time practices. Based on action research with caseworkers to trial, observe and reflect on new ways of connecting children with birth relatives using digital technology, this study found these changes encouraged caseworkers to focus on and strengthen the interactions among children and birth relatives and carers. The results extend beyond how to cope with future lockdowns and offer lessons about how to support meaningful relationships among children and their two families. This includes practical, emotional and instrumental support such as resources for families who lack hardware, software or technology skills.

## CONFLICT OF INTEREST

The authors have no conflict of interest to declare.

## Data Availability

Research data are not shared.
